# Annual Meeting of Constituents

**Published:** 1916-12-30

**Authors:** 


### THE METROPOLITAN HOSPITAL SUNDAY FUND.

### Annual Meeting of Constituents.

The annual meeting of constituents of the Fund was held at the Mansion House on Thursday, December 21, under the presidency of the Lord Mayor (Sir William Dunn).

The Secretary (Mr. Arnold James) announced that among the regrets for absence were those from the Bishop of Islington, Sir Henry Burdett, the Rev. Frank James, Sir Marcus Samuel, and Dr. Parker Young.

**The Lord Mayor,** having read to the meeting the law governing the conduct of the annual meetings, said he was very pleased that one of his first functions at the Mansion House—and he did not doubt that with his predecessors in the office it was an equal pleasure—was to preside at a meeting of the Hospital Sunday Fund. He, like those present, had known this Fund for many years, and was therefore able to testify to the great and noble work it was carrying out. He believed it was during the mayoralty of Sir Sydney Waterlow that the institution was started, and though it was not his original idea, he gave effect to it, and he believed that all Sir Sydney's successors had taken the chair at the meetings of the Fund. It was needless for him to say how many funds were in operation in this time of war, and for these the Mansion House was continually being besieged. As Lord Mayor of London he sincerely hoped the Metropolitan Hospital Sunday Fund would not suffer. It was so firmly established, and he believed the people believed so thoroughly in it, knowing the good work it did, that it would continue to flourish. Owing to a good deal of poverty which prevailed, he hoped the takings would be even greater than before.

The annual report was taken as read.

**Sir Ernest Tritton,** in proposing its adoption, expressed the pleasure which the constituents felt in having the Lord Mayor in the chair. They would wish him a very happy year of office, and, above all, they hoped that during his year peace might be once more established in the world. The Council considered that, bearing in mind the times through which the country was passing, and the many charitable appeals being made, the figure reached, though £4,900 less than last year, would be regarded as satisfactory. At any rate, the expenses had not increased, and he hoped constituents would feel that the matter had been worked satisfactorily. Considering all the circumstances, he thought it a wonderful thing that the Fund was able this year to distribute to the various hospitals and charities £70,000. The people of London loved their hospitals, and never more than at the present time, when within their walls there were so many gallant sick and wounded sailors and soldiers, who had been brought back to the Old Country after exhibiting a heroism and an endurance unsurpassed in the annals of the world. Speaking as the vice-chairman, he could say the Council was extremely grateful for the earnest and faithful advocacy of the claims of the Fund in the various pulpits, and they looked forward to the future with confidence that the collections for the 1917 distribution would show no diminution on this year's figure, but rather a gratifying increase. The hand of Death had been busy in the ranks of the Council of the Fund, for during the past year it had lost Sir Richard Biddulph Martin, who was one of the founders, and was Honorary Secretary of the Fund when it started. Also Sir Walter Vaughan Morgan, who had attended the meetings of the Fund for many years. They had also lost Mr. F. Henry Norman, who served on the Committee of Distribution for thirty years, and had rendered it most valuable services. Another loss was that of Prebendary Anderson, who for some time was chairman of the General Purposes Committee, and a very keen supporter of the Fund. Members of the Council missed those gentlemen, and they would miss them increasingly as time went on. However, the meeting would be electing gentlemen to take their places who would be full of zeal for the good work in hand. The Fund had further lost a most generous donor in Mr. William Herring, who for many years gave them £1,000, and latterly £500 a year. He also would be very greatly missed.

**The Rev. Prebendary Reynolds** seconded the resolution, and it was unanimously adopted.

**The Rev. Dr. Carter** proposed and the **Rev. Mr. Stanway** seconded the re-election of the retiring members of Council, with the addition of the Bishop of Chelmsford, the Rev. Frederick Rice, M.A., the Rev. Prebendary Reynolds, M.A., the Rev. Bernard J. Snell, M.A., the Hon. Edward Dudley Ryder, the Hon. Sydney Edward Marsham, Robert Martin-Holland, Esq., C.B., and Stuart de la Rue, Esq., to fill vacancies, which was carried.

### A Fixed Date for Hospital Sunday.

**The Archdeacon of London** proposed: "That the recommendation of the Council that in future Hospital Sunday be an annual fixture for the fourth Sunday in June, be and is hereby adopted, and accordingly: (*a*) That the date of Hospital Sunday 1917 be June 24, and (*b*) that in Law V. of the Constitution 'July 31' be substituted for 'June 30.'"

As chairman of the General Purposes Committee, he wished to say that they sent this final recommendation up to the Council after very considerable discussion. There were two points before them. First, whether Hospital Sunday should be a fixed day; and, secondly, if so, what day should it be? It was decided that it should be a fixed day, and the settlement of the second point was a more difficult matter, for the day which was agreeable to one completely failed to suit another. After much debate and trouble, they decided by a majority on the fourth Sunday in June, as that appeared to present, on the whole, the least difficulty: it seemed to meet most of those who wished to get the greatest benefit for the Fund. When the resolutions were presented to the Council, exactly the same thing happened, and it was agreed that more pitfalls were avoided in the General Purposes Committee's recommendations than in any other. Obviously, if there should be such a great and unforeseen event as thanksgiving for peace, it would be in the power of the Council to change the date.

**The Rev. W. Hardy Harwood,** in seconding the resolution, said that if there were difficulties associated with a fixed day, obviously there were greater ones about a movable day. Scarcely a year had passed when a group of Churches found they could not keep the day appointed for the purpose, and asked that they might take their collection for the Fund on some other Sunday. Therefore, in accepting the present recommendation, they would not be plunging into new difficulties. Canon Fleming told him that he found when people were out of town they answered the written appeal, of which he sent out many, by sending a cheque for a definitely larger amount than they were likely to have put into the plate if they had been at church.

The motion was carried unanimously.

**Sir Dyce Duckworth** proposed a cordial vote of thanks to the Lord Mayor for presiding. Those who had been connected with this movement for many years realised that the presence of the Lord Mayor in the chair was one of its great motor powers, and it had certainly been so, without a single exception that he could recall. The presence of the Lord Mayor was one of the great assets of the organisation, and he did not doubt that, under the presidency of Sir William Dunn, the collection for next year would not compare unworthily with that announced as having been obtained this year.

The resolution was carried by acclamation.

**The Lord Mayor,** in reply, assured the meeting it was a great pleasure to him to preside. He hoped he would be able to attend the Service at St. Paul's on June 24, when he hoped to see also many of those who had come to this meeting. He was very anxious that this year the Fund should be a great success, and anything which he could do, in his small way, would be a great delight to him.

